# MicroRNA-155: Regulation of Immune Cells in Sepsis

**DOI:** 10.1155/2021/8874854

**Published:** 2021-01-08

**Authors:** Ming Chen, Fuquan Wang, Haifa Xia, Shanglong Yao

**Affiliations:** ^1^Department of Anesthesiology, Union Hospital, Tongji Medical College, Huazhong University of Science and Technology, Wuhan 430022, China; ^2^Institute of Anesthesia and Critical Care Medicine, Union Hospital, Tongji Medical College, Huazhong University of Science and Technology, Wuhan 430022, China

## Abstract

MicroRNAs are small noncoding RNAs which regulate gene expression at the posttranscriptional level. miR-155 is encoded by the miR-155 host gene (miR155HG), also known as the noncoding B cell integration cluster (BIC). MicroRNAs are widely expressed in various hematopoietic cells and are involved in regulating the immune system. In this review, we summarized how miR-155 modulates specific immune cells and the regulatory role of miR-155 in sepsis. miR-155 is expressed by different populations of innate and adaptive immune cells and is involved in the regulation of development, proliferation, and function in these cells. Sepsis is associated with uncontrollable inflammatory responses, accompanied by unacceptably high mortality. Due to the inadequacy of diagnostic markers as well as treatment strategies, treating sepsis can be a huge challenge. So far, a large number of experiments have shown that the expression of miR-155 is increased at an early stage of sepsis and that this increase is positively correlated with disease progression and severity. In addition, by blocking the proinflammatory effects of miR-155, it can effectively improve sepsis-related organ injury, providing novel insights to identify potential biomarkers and therapeutic targets for sepsis. However, since most of the current research is limited to animal experiments, further clinical research is required to determine the function of miR-155 and its mechanism related to sepsis.

## 1. Introduction

MicroRNAs (miRNAs) are a class of small noncoding RNAs which are involved in RNA silencing and posttranscriptional regulation of gene expression [1]. A single miRNA can target several genes, while different miRNAs can also regulate the same target gene. miRNAs regulate the expression of genes through binding to the 3′ untranslated regions (UTRs) of target mRNAs [2]. Through these regulatory effects, miRNA has been proven to be essential for immune responses and is involved in autoimmune diseases and cancers [3], suggesting its potential utility as diagnostic markers or therapeutic targets in this disease.

miR-155 is a microRNA that is derived from the noncoding RNA transcript of the protooncogene B cell integration cluster (BIC) [4]. The sequence of miR-155 between humans and mice is conserved [5], making it an attractive target for transforming mouse research into human clinical application. miR-155 has been proven to be highly specific for hematopoietic cells [6], suggesting that it may participate in the human immune system.

miR-155 can be induced by specific transcription factors such as AP-1, MYB, PU.1, STAT3, and Ets2 [7–11]. Through the AP-1-mediated mechanism, the expression of miR-155 is highly induced by TLR agonist activation [4, 12]. miR-155 can target multiple inflammatory mediators including TNF-*α* and interferons [11]. In contrast, the anti-inflammatory cytokine IL-10 suppresses the TLR4 signaling and downregulates miR-155 in a STAT3-dependent manner [13]. Besides, IL-10 can inhibit the transcription of Ets2, thus leading to the suppression of miR-155 in response to LPS [10].

Bioinformatics analysis of human miR-155-5p mRNA targets using the TargetScanHuman release 7.1 database (http://www.targetscan.org/vert_71) revealed the presence of at least 552 transcripts with conserved sites, containing a total of 592 conserved sites and 239 poorly conserved sites. It can be speculated that the miR-155-3p may also have potential to target thousands of mRNA which have not been identified as its potential target. miR-155 encodes many key regulatory proteins related to human pathological and physiological processes, and some of the target genes have been experimentally confirmed (Table 1).

## 2. Roles of miR-155 in Immune Cell Development and Function

**Figure 1:**
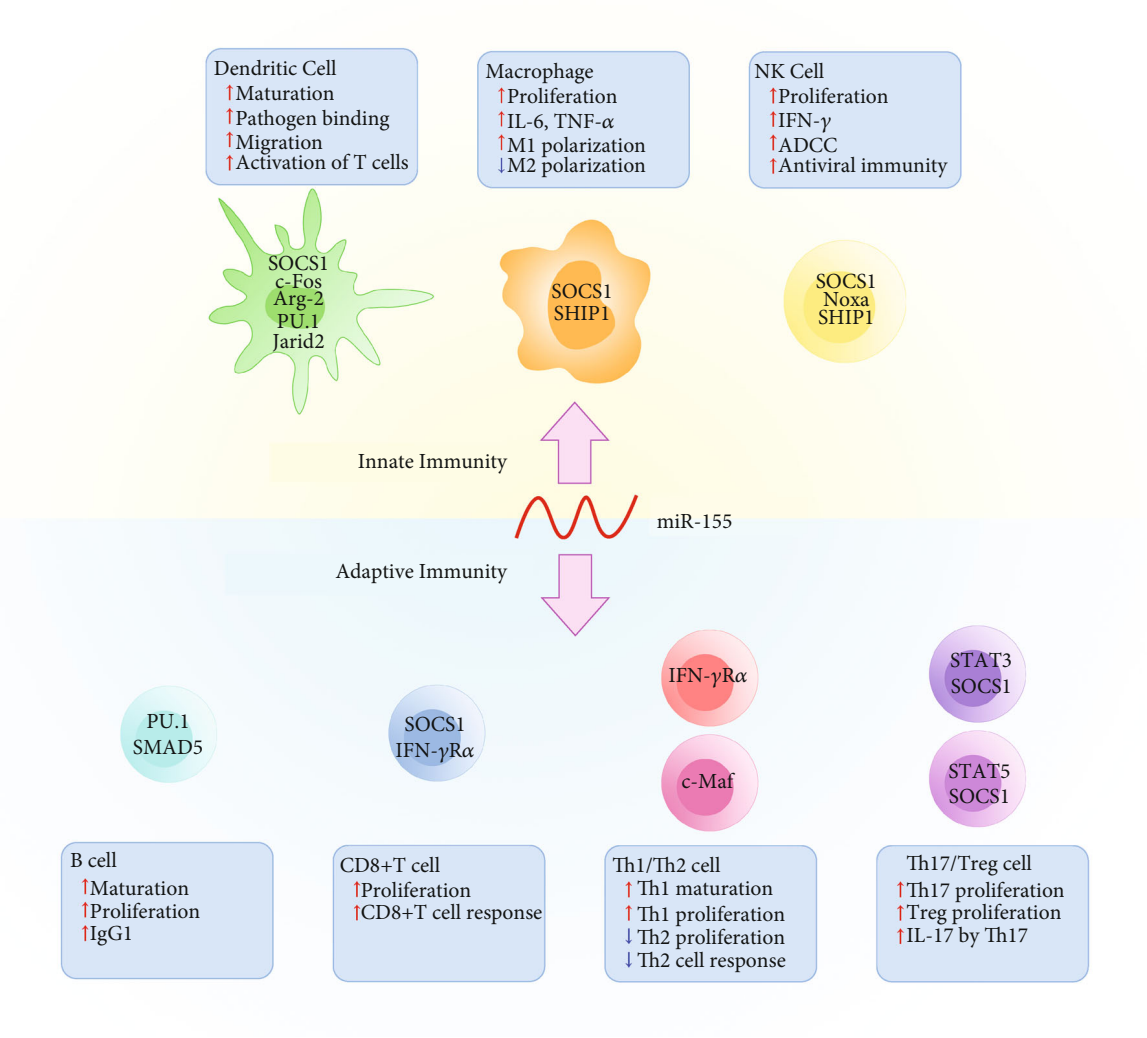
Roles of miR-155 in immune cell development and function. miR-155 and dendritic cells. ADCC: antibody-dependent cytotoxicity; Th1: T helper 1; Th2: T helper 2; Th17: T helper 17; Treg: regulatory T.

miR-155 is highly expressed by a broad range of cell types in the innate immune system as well as adaptive immune system including monocytes, macrophages, dendritic cells, B cells, and T cells [14–18]. Moreover, miR-155-deficient mice showed impaired function of B and T lymphoma cells as well as dendritic cells (DCs) [19], suggesting that miR-155 is essential for maintaining immune homeostasis (Figure 1).

### 2.1. miR-155 and Dendritic Cells

The myeloid lineage-derived dendritic cells are dedicated to the uptake and presentation of antigen and thus initiate adaptive immune responses. miR-155 is strongly induced in multiple mouse and human DC subsets, and miR-155 expression could be a decisive feature of DC maturity as inhibition of miR-155 targets (SOCS1, c-Fos, and Arg-2) is essential for the expression of MHC class II and costimulatory molecules [20–23]. However, the number of conventional DCs (cDCs) and plasmacytoid DCs (pDCs) was unaffected in miR-155 knockout mice, which means miR-155 does not contribute to DC development [22]. As for the impact on DC function, research showed that miR-155 targets the transcription factor PU.1, thereby inhibiting the expression of DC-specific intercellular adhesion molecule-3 grabbing nonintegrin (DC-SIGN) [21]. miR-155 regulates DC-induced activation of CD8+ T cells through downregulation of SHIP1 [24] as well as controls DC-CD4 T cell interaction [25]. Moreover, miR-155 plays a key role in mature DC migration to the T cell area of draining lymph nodes by targeting Jarid2 [26].

### 2.2. miR-155 and Macrophages

Macrophages are critical for the initiation of adaptive immune response in the early phase of infection. As a highly heterogeneous cell population, macrophages can adapt their functions in response to various microenvironmental signals and thus have distinct functions [27]. In general, macrophages differentiate into two groups: M1 macrophages have a proinflammatory and cytotoxic function through the induction of T helper type 1 (Th1) cell responses, whereas M2 macrophages participate in Th2-type responses which are anti-inflammatory [28]. miR-155 can promote the proliferation of macrophages and the secretion of proinflammatory cytokines such as IL-6 and TNF-*α* by targeting SHIP1 and SOCS1, respectively [29]. In addition, SOCS1 targeted by miR-155 negatively regulates M1 macrophage polarization via suppressing the interferon-*γ*-induced JAK2/STAT1 pathway and TLR/NF-*κ*B signaling [30, 31]. Besides, miR-155 directly targets IL-13 receptor *α*1 (IL13R*α*1) leading to diminished activation of STAT6 and M2 polarization [32]. Consequently, accumulation of miR-155 leads to M1 polarization but inhibits M2 polarization.

### 2.3. miR-155 and Natural Killer Cells

Natural killer (NK) cells are specialized in recognition and killing of abnormal host cells such as tumor cells or those infected with pathogens [33]. Upregulation of miR-155 increased the number of NK cells, enhanced the function of secreting IFN-*γ*, and significantly improved antibody-dependent cytotoxicity (ADCC) by reducing the expression of SHIP1 and enhancing the activation of ERK and AKT kinases [34]. Another research indicates that miR-155 reduced the expression of Noxa and SOCS1 in NK cells, thereby enhancing antiviral immunity in NK cells [35].

### 2.4. miR-155 and B Cells

B cells are important immune cells and have the ability to differentiate into antibody-secreting plasma cells as well as regulate immune responses via cytokine secretion and antigen presentation [36]. After being activated by antigen encounters and helper T cells, B cells migrate to the center of the follicle to form a germinal center (GC), where they rapidly proliferate and undergo somatic hypermutation, affinity maturation, and antibody class switching to generate ultimately long-lived memory B cells and terminally differentiated antibody-producing plasma cells [37]. miR-155 was found to be critical in the development of B cells by targeting SMAD5 and modulating the transforming growth factor *β* (TGF-*β*) pathway [38, 39]. Tumor necrosis factor (TNF) and lymphotoxin (LT) signaling are essential for germinal center formation. miR-155 affects B cell maturation steps by regulating cytokine production or targeting different transcription factors [40]. In miR-155 knockout mice, B cells lacked TNF and LT-*α* production and showed reduced antigen-specific immunoglobulin G1 (IgG1) antibody titer and GC B cell fraction indicating that miR-155 modulates B cell maturation and function [17]. The regulation of immunoglobulin class-switched plasma cells by microRNA-155 may be achieved through targeting PU.1 as overexpression of PU.1 in wild-type B cells impaired the emergence of IgG1-switched cells [41].

### 2.5. miR-155 and T Cells

T cells differentiate into CD4+ and CD8+ cells in the thymus. CD4+ cells can differentiate into at least four distinct subsets, including T helper 1 (Th1), T helper 2 (Th2), T helper 17 (Th17), and regulatory T (Treg) cells, each with different functions.

CD8+ T cells or cytotoxic T lymphocytes (CTLs) can recognize and kill virus-infected cells and tumor cells [42]. Interestingly, primary effector and effector memory CD8+ T cells express a low level of miR-155 while effector memory cells express an intermediate level of miR-155, suggesting that the amount of miR-155 expression may regulate the response capacity of CD8+ T cells at different stages of differentiation [16, 43]. The lack of miR-155 results in upregulated type I interferon signaling and reduced CD8+ T cell proliferation [16]. In terms of immune function, miR-155 deficiency greatly attenuates the function of CD8+ T cell response to viral and bacterial infections [16]. Targeting SOCS1 by miR-155 enables antiviral response and cytokine signaling in effector CD8+ T cells [15]. Therefore, miR-155 may have important implications for immune function of CD8+ T cells.

Th1 cells mediate cellular immunity-targeted intracellular pathogens and autoimmunity by producing IL-2, IFN-*γ*, and TNF-*β*, while Th2 cells participate in humoral immunity and allergy by secreting IL-4, IL-5, IL-9, and IL-13 [44, 45]. Under nonpolarizing conditions, miR-155-deficient T cells tend to Th2 differentiation [17]. Corresponding to this, miR-155 decreases sensitivity of Th1 cells to the antiproliferative effects of IFN-*γ* [46] and enhances Th1 maturation by suppressing IFN-*γ*R*α* expression [47]. c-Maf as a Th2-specific transcription factor induces Th2 differentiation by a mechanism that is IL-4-dependent and inhibits the differentiation of Th1 cells by a mechanism that is independent of Th2 cytokines [48]. miR-155 diminishes the level of c-Maf in CD4+ T cells and results in the attenuation of Th2 cell responses [19].

Treg cells expressing specific transcription factor FoxP3 inhibit effector T cell functions by secreting immunosuppressive cytokines such as IL-10 and TGF-*β* or by inhibition of immune checkpoint receptors including TIGIT and CTLA-411 [49]. Th17 cells secrete IL17A, IL-17F, IL-21, IL-22, and CCL20; express the characteristic transcription factor ROR*γ*t; and promote inflammation in response to infection [49]. Treg and Th17 cells do not function in isolation, and the imbalance between the two may play a vital role in the progress of a large number of human diseases [50]. SOCS1 negatively regulates the development of Th17 and Treg cells through inhibiting transcription factors STAT5 and STAT3 [30]. Through downregulating SOSC1, miR-155 induces Treg proliferation and expression of FoxP3 without affecting IL-10 and TGF-*β* secretion [51]. This indicates that miR-155 can regulate Treg proliferation, but it does not seem to directly influence the immunosuppressive function of Treg. Moreover, another study shows that proliferation of miR-155-knockout Treg cells was not much affected when they were reintroduced into a noncompetitive lymphopenic environment [52]. These results suggested that miR-155 is necessary to maintain the proliferation of Treg cells under the condition of a limited amount of growth factors [53]. Similarly, miR-155 promotes the generation of Th17 cells via targeting SOCS1 [54]. However, miR-155 induced the secretion of IL-17 by Th17, which proved that miR-155 improves the proinflammatory function of Th17 cells [54, 55]. Given that miR-155 enhances the secretion of IL-17 by Th17 cells without affecting immunosuppressive function of Treg, it may play a role in regulating inflammatory diseases through the targeting of SOCS1.

## 3. The Role of miR-155 in Sepsis

Since miR-155 plays a wide range of regulatory roles in immune cells, it is not surprising that miR-155 is closely involved in the regulation of inflammatory disorders. Recently, it is reported that the hemorrhage-induced cerebrospinal fluid miR-155 is expected to be a biomarker of intraventricular hemorrhage in premature infants as miR-155 effectively reflected inflammatory conditions [56]. miR-155 is also closely related to the regulation of autoimmune diseases, such as rheumatoid arthritis (RA) [57], systemic lupus erythematosus (SLE) [58], and experimental autoimmune encephalomyelitis (EAE) [59]. As for viral infections, miR-155 is required for an optimal CTL response by affecting CD8+ T cells [15]. Besides, miR-55 was found to play a novel role during viral respiratory infections in young children by promoting Th1 polarization [60].

Sepsis is a common disease with high mortality and has become the leading cause of death in intensive care units (ICUs). More than 25-30% of patients with sepsis die of the disease, and the hospital mortality rate for septic shock is close to 40-60% [61]. Due to the high mortality and morbidity of sepsis, the key to improve the survival rate of sepsis patients is to diagnose sepsis at an early stage, accurately evaluate the severity of sepsis patients, and select appropriate treatment methods. Therefore, it is very important to find biomarkers for the early diagnosis of sepsis. The laboratory indexes of the clinical diagnosis of sepsis include C-reactive protein (CRP), procalcitonin (PCT), and IL-6 [62]. As a single biomarker has limited value, the use of new biomarkers and the combination of several markers is recommended in improving diagnostic accuracy [63, 64]. At present, the mortality of sepsis remains unacceptably high even if a variety of treatment methods are widely used including antimicrobial therapy, source control, fluid resuscitation, and mechanical ventilation [65]. Due to the lack of specific diagnostic markers and treatment strategies, as well as the clinical heterogeneity of sepsis, it may become a huge challenge to treat sepsis [66].

Sepsis is a life-threatening organ dysfunction that is caused by dysregulated host responses to infection [67]. The occurrence and development of sepsis are the result of imbalance between the proinflammatory and anti-inflammatory mechanisms in the body. Innate immune cells including monocytes and macrophages express pathogen recognition receptors (PRRs) including Toll-like receptors (TLRs), especially TLR4, that recognize pathogen-associated molecular patterns (PAMPs) leading to the activation of nuclear factor *κ*B (NF-*κ*B) signaling pathways, which can lead to inflammation driven by proinflammatory cytokines such as TNF-*α*, IL-1*β*, and IL-6 [68]. The adaptive immune response is mainly mediated by T lymphocytes and B lymphocytes. After activation, CD4+ Th1 and Th17 cells secrete proinflammatory cytokines such as interferon-*γ* (IFN-*γ*), interleukin-17 (IL-17), and granulocyte macrophage colony-stimulating factor (GM-CSF) while Th2 and Treg cells secrete anti-inflammatory cytokines IL-4 and IL-10 to prevent excessive inflammatory response [69]. When these two reactions are unbalanced, a large amount of inflammatory mediators will be released" instead, resulting in organ damage or even death. Therefore, the key to the treatment of sepsis is to control the immune inflammatory response and prevent the progression of the inflammatory cascade. Unfortunately, during the last years, the anti-inflammatory treatment aimed at inhibiting the acute inflammatory response using glucocorticoids, nonsteroidal anti-inflammatory agents, and anti-TNF-*α* antibodies failed in achieving significant results [70]. Due to the unclear regulatory mode, researches based on the mechanism of immune inflammation have not yet screened out effective treatment strategies for sepsis.

### 3.1. miR-155 as a Biomarker of Sepsis

miRNAs have biological characteristics such as small molecules, simple structures, high specificity and selectivity, and stable existence in circulation that can be extracted and detected. Moreover, miRNAs are widely present in serum, plasma, and other body fluids, which make them convenient to determine the levels in the clinic [71]. Nanopore-based techniques have been recently developed for the rapid detection of miRNAs, which give miRNAs a promising advantage in time compared with bacteriological culture. Many researchers have tried to use it as a potential organism for disease diagnosis. Due to the inadequacy of diagnostic markers and treatment strategies, as well as clinical heterogeneity, sepsis becomes a major challenge. Hence, miRNAs may serve as potential biomarkers in the diagnosis of sepsis and may act as prognostic parameters. In particular, miR-155 was confirmed to be significantly elevated in sepsis patients than in healthy controls by a number of studies [72–75].

Analysis of miRNA expression revealed that the expression of miR-155 was related to severity and prognosis of sepsis. TLRs are critical for initiating the systemic inflammatory response and contributing to organ dysfunction in sepsis [76]. DNA-microarray analyses revealed that when TLR signaling pathways were activated, the expression levels of miR-155 in human as well as murine initial CD4+ T cells and Treg cells increased [77]. The correlation between miR-155 and sepsis can be seen in animal experiments. In the model of LPS-induced sepsis, the level of miR-155 was significantly elevated in alveolar macrophages and reached a peak 6 hours after LPS injection, suggesting that it may be of certain value in the diagnosis of early sepsis [78]. In agreement with it, there were significantly upregulated miR-155 in both lung and spleen tissues after mice were administrated with LPS [79]. Following sepsis caused by cecal ligation and puncture (CLP), an increase in miR-155 was found in the serum of mice [76]. A study which included 60 sepsis patients and 30 healthy controls indicated that the expression of miR-155 in sepsis patients significantly increased compared to healthy controls [74]. In addition, the level of miR-155 was positively associated to a higher SOFA score and a greater severity of sepsis [74]. The clinical accuracy of miR-155 in predicting the 28-day survival of sepsis patients by the receiver operating characteristic (ROC) curve has a satisfactory clinical efficacy [74]. Another clinical research indicated that the level of miR-155 in the myocardium and plasma was significantly upregulated in human sepsis [80]. Among ICU-admitted sepsis patients, nonsurvivors had higher miR-155 plasma levels than survivors [80]. Notably, a clinical study suggests that elevated miR-155 has a potential clinical relevance in sepsis-related cardiac dysfunction [81]. In addition, the circulating miR-155 obviously decreased at 48 hours after admission, indicating that the upregulation of miR-155 was limited to the early stage of human septicemia [80]. Thus, the analysis of circulating miR-155 may serve as a diagnostic biomarker in sepsis and contribute to the rapid initiation of treatment.

### 3.2. miR-155 as a Potential Therapeutic Target in Sepsis

**Figure 2:**
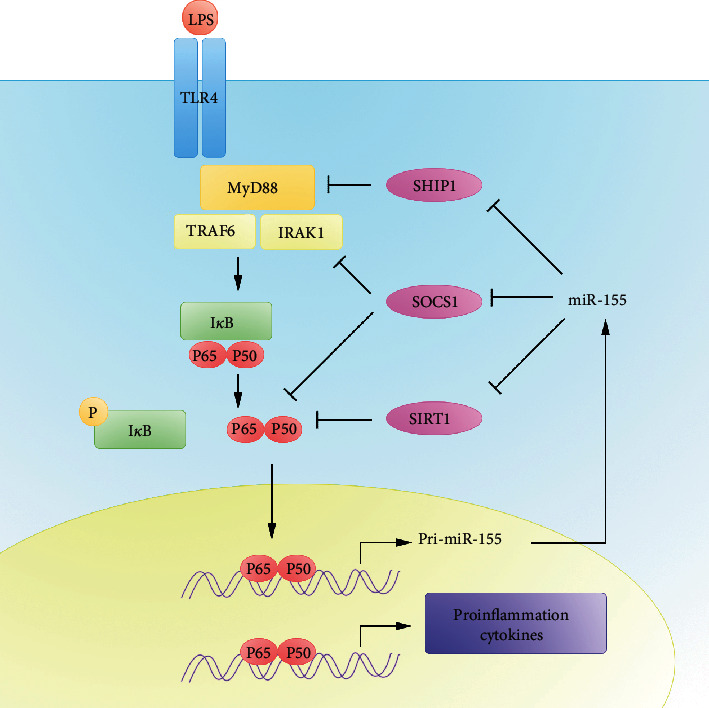
miR-155 modulates TLR4 signaling pathway in sepsis.

As pointed out, the TLR4-induced NF-*κ*B signaling pathway involves in the inflammatory response in sepsis. It has been proven that SHIP1 and SOCS1 help restrain proinflammatory signals through this pathway as negative regulators [30, 82]. miR-155 can target and inhibit the expression of SHIP1 and SOCS1; thus, it may promote the inflammatory response of sepsis (Figure 2). Indeed, serum exosome-derived miR-155 from sepsis mice promote macrophage proliferation and production of proinflammatory cytokines by targeting SHIP1 and SOCS1 [83]. After bacterial infection, miR-155, which is highly expressed by monocytes and macrophages, can downregulate the expression of the target gene SHIP and promote the activation of the PI3K/Akt signaling pathway and the secretion of proinflammatory cytokines [84]. In addition, TNF-*α* is thought to be an important mediator in the development of endotoxin shock and multiple organ dysfunctions. Studies showed that TNF production by miR-155-deficient B cells was significantly reduced compared to that by normal controls [17], whereas miR-155 overexpressed mice produced higher levels of TNF-*α* in response to LPS and were more sensitive to LPS-induced endotoxin shock [85]. IL-10, a vital anti-inflammatory cytokine, presents protective effects in sepsis [86]. The levels of IL-10 were obviously higher in miR-155-knockout mice after LPS administration compared to WT mice, confirming that miR-155 inhibits IL-10 production and then mediates the dysregulation of inflammatory response [87]. Besides, miR-155 suppressed the LPS-induced IL-10 expression in B lymphocytes [88].

miR-155 is also involved in sepsis-related organ damage and complications. The lung is the first organ to be damaged and plays a vital role when sepsis occurs. Almost half of septic shock patients have acute lung injury (ALI) [89]. Many studies have pointed out that the reduction of miR-155 protects against acute lung injury induced by sepsis. In acute systemic inflammatory responses such as sepsis, SIRT1 involves in resolution of inflammation by negatively regulating NF-*κ*B pathways [90]. Consistent with this, in CLP-induced ALI mice, the miR-155 inhibitor notably improved the survival rate in CLP mice, receded pathological changes in the lung tissues, attenuated the expression of TNF-*α* and IL-1*β* in the serum, and decreased neutrophil infiltration and pulmonary edema by targeting SIRT1 [91]. Moreover, miR-155 directly targeted HIF-1*α* and thus suppressed the anti-inflammatory protein HO-1, preventing a positive effect on LPS-induced cell injury and inflammation response in ALI after LPS administration [92]. Isoflurane significantly inhibited the expression of miR-155 and alleviated LPS-induced ALI by targeting signaling through miR-155, HIF-1*α*, and HO-1 [92]. Another study demonstrated that the deficiency of miR-155 inhibited the expression of Ang-2 and apoptosis-associated caspases-3 and -9, owning to the phosphorylation of MLC in the lungs [93]. As a result, the severity of lung injury decreased in the miR-155-deficient mouse model of ALI [93]. In sepsis patients, myocardial miR-155 expression is positively correlated with the expansion of interstitial regions [80]. miR-155 knockout and pharmacologic inhibition significantly improved sepsis-associated cardiovascular dysfunction (SCVD) and mortality in two experimental sepsis models, suggesting the potential value of miR-155 treatment in septic shock patients [80]. A study attempted to antagonize miR-155 in septic mice through administration of the miR-155 antagomir [94]. The result demonstrated that the miR-155 antagomir inhibited oxidative stress, mitochondrial dysfunction, cell apoptosis, and ER stress by targeting Nrf-2, thereby reducing septic liver injury [94]. In line with this, MCP-1-induced protein 1 (MCPIP1) downregulates miR-155 and thus inhibits NF-*κ*B signaling by attenuating the production of proinflammatory cytokines, modulating LPS-induced inflammation and liver injury in vivo as well as in vitro [95]. In terms of sepsis-associated kidney injury, the miR-155 inhibitor alleviates LPS-induced kidney injury by decreasing the levels of TNF-*α* and IL-6 in renal tissues and the mortality of LPS-treated mice, indicating that miR-155 may be a potential therapeutic target for treatment of sepsis-related kidney injury [96].

miRNA antagonists are synthetic-modified oligonucleotides complementary to the endogenous miRNAs, designed to inhibit the function of miRNAs. The satisfactory results of past studies show that miRNA-based therapeutics is promising in clinical medicine [97]. Current strategies to inhibit miRNAs include the use of antagomirs, antisense oligonucleotides (AMOs), locked nucleic acid (LNA) anti-miRNAs, miRNA sponges, and miRNA masks [98]. Antagomir is a widely studied anti-miRNA molecule which is considered to be highly efficient and long-lasting [99]. By inhibiting miR-155, the miR-155 antagomir relieved septic liver injury [94]. Another study showed that the miR-155 antagomir improved cardiac function in LPS-induced sepsis [81]. LNA anti-miRNAs are chemically modified oligonucleotides with increased stability in vivo following several modifications [100]. In one study, the inhibition of miR-155 in an LPS-induced sepsis mouse model by systemically administered LNA anti-miRNAs resulted in attenuated proinflammatory response [101]. However, there are issues about miR-155 antagonists that need to be addressed for clinical applications. Systemic miRNA inhibition may induce side effects [102]. Besides, it is a significant challenge to deliver miRNA antagonists to specific cells. Therefore, strategies should be adopted to reduce the side effects mediated by off-target gene silencing and improve the efficiency.

## 4. Summary and Prospect

In this review, we discuss the vital role of miR-155 in immunobiology and sepsis. Due to its important role in various immune processes, miR-155 has been extensively studied and reported in the field of sepsis progression, prognosis, and treatment. So far, various targets of miR-155 have been identified, suggesting that miR-155 may have potential value in the diagnosis and treatment of sepsis. As most of the current research is limited to animal experiments, further clinical studies are necessary to shed light on the role of miR-155 in prognostic and therapeutic potential.

If we can figure out the specific mechanism of miR-155 to regulate the LPS signaling pathway and find the target genes it acts on, then when the body has an uncontrolled inflammatory response such as sepsis, we can reduce excessive inflammation and alleviate the tissue and organ damage caused by sepsis. This will provide new methods and new approaches for the diagnosis and treatment of sepsis and other inflammation-related diseases.

## Figures and Tables

**Table 1:** Targeted genes of miR-155 that have been experimentally confirmed.

No.	Target gene	Function
1	SOCS1	Regulator of cytokine signal transduction
2	CEBPB	Related to the regulation of proinflammatory cytokines during macrophage activation and the acute phase response
3	TP53INP1	Proapoptotic stress-induced p53 target gene
4	INPP5D/SHIP1	Negative regulator of cell proliferation and survival
5	FOXO3	Tumor suppressor
6	BACH1	Transcription regulator protein
7	AGTR1	Mediates the major cardiovascular effects of angiotensin II
8	TAB2	Activator of MAP3K7/TAK1
9	SMAD2	Mediates the signal of the transforming growth factor- (TGF-) *β*
10	SMAD5	Modulates signals of bone morphogenetic proteins (BMPs)
11	JARID2	Organ development
12	SPI1/PU.1	Myeloid and B-lymphoid cell development
13	ETS1	Related to the differentiation and development of immune cells
14	E2F2	Regulation of cell cycle and tumor suppressor protein
15	SIRT1	Cellular regulation related protein deacetylating enzyme
